# Policy Series: The Elder Justice Act, Nursing Home Reform, and Funding Legislation

**DOI:** 10.1093/geroni/igaa057.2546

**Published:** 2020-12-16

**Authors:** Brian Lindberg

## Abstract

This session will provide updates on major federal efforts to address elder abuse, neglect, and exploitation, including strategies for prevention, intervention, services, and prosecution. Congress has been working on both reauthorizing the Elder Justice Act and policies to address poor long-term care facility quality issues, and this panel will provide an update on those efforts and what lies ahead in2021. The panel will include elder justice and nursing home advocates and congressional staff.

